# A reproducible method to generate multi-building, multi-climate HVAC operation datasets with a stochastic exploratory controller

**DOI:** 10.1016/j.mex.2026.103866

**Published:** 2026-03-18

**Authors:** Ferran Aran Domingo, Pablo Fraile Alonso, Josep Rius Torrentó, Oriol Agost Batalla, Ignasi Barri Vilardell, Jordi Vilaplana Mayoral, Jordi Mateo Fornés

**Affiliations:** a GFT Technologies S.L.U., Parc Agrobiotech Lleida Parc de Gardeny, Turó de Gardeny, Edifici H1 25003 Lleida, Spain; b Department of Computer Science, University of Lleida, Carrer de Jaume II, 69 25001 Lleida, Spain

**Keywords:** HVAC, Building energy systems, Dataset generation method, Sinergym, BOPTEST, EnergyPlus, Modelica, Exploratory control, Transfer learning, Digital twins

## Abstract

Building control research increasingly requires datasets that are reproducible, controllable, and rich in action–state coverage. We present a method to generate multi-year HVAC operation time series across heterogeneous buildings and climates using open-source building simulation frameworks, which expands control diversity using a deliberately stochastic supervisory controller. The workflow combines EnergyPlus-based simulation via Sinergym for multi-building/multi-climate “source” domains and Modelica-based simulation via BOPTEST for a distinct “target” domain to support transfer-learning evaluation and reproducible comparisons. Alongside the default rule-based controller (RBC), we implement a stochastic exploratory policy that interleaves stochastic drift, ramps, oscillations, jumps, and noisy holds to produce non-routine heating/cooling setpoint trajectories under operational bounds. The method produces standardized 15-minute multivariate time series including indoor temperature, outdoor weather, setpoints, and HVAC power, and releases both the datasets and the full code needed to reproduce or extend them.

• Reproducible pipeline combining Sinergym (EnergyPlus) and BOPTEST (Modelica) under a common interface.
• Stochastic HVAC supervisor that broadens setpoint distributions beyond standard schedules.
• FAIR release of code + datasets to enable evaluation and reproducible comparisons, transfer learning, and robustness studies.

## Graphical abstract

**Figure:**
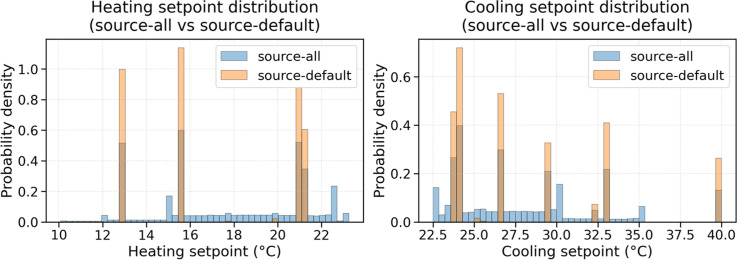

**Specifications table**

**Table:** 

**Subject area**	Computer science
**More specific subject area**	Energy systems, HVAC control
**Name of your method**	Rich action-state multi-building multi-climate HVAC dataset generation with stochastic supervisory control
**Name and reference of original method**	None
**Resource availability**	Source code: https://doi.org/10.5281/zenodo.18378593 Datasets: https://doi.org/10.57967/hf/7507

## Background

Existing public datasets on building operation cover a range of building types and variables. For example, the AlphaBuilding dataset [1] provides 1,395 one-year EnergyPlus [2] simulations of a medium office building across three climates (Miami, San Francisco, Chicago) at 10-minute resolution, including HVAC, lighting, occupancy, and energy-load data. As for real-building datasets, LBNL Building 59 [3] and HouseZero [4] offer three and two years of detailed office-building operation data, respectively, capturing indoor conditions, HVAC performance, and energy use under rule-based control. Larger multi-building datasets such as the Building Data Genome Project [5] provide hourly data for over 1,600 non-residential buildings worldwide, covering electricity, thermal, and water usage.

Various datasets explicitly target control or RL research. The CityLearn Challenge [6] provides synthetic environments for evaluating learning-based control strategies. B2RL [7] is the most relevant precedent by providing simulated building responses produced under learning policies, offering some exploratory trajectories. However, it remains limited to a single simulated building and is primarily designed for offline RL evaluation and reproducible comparisons. The Smart Buildings Control Suite [8] includes six years of real operational data from 11 buildings along with calibrated digital-twin simulators. Finally, the CLUE project [9] publishes data from a five-zone simulated building which the authors used to train building-control models in that same study. While these datasets are highly valuable for comparisons, none combine multi-building, multi-climate simulation coverage with explicitly stochastic control policies.

Recent work has applied modern deep learning and foundation-style models to occupancy-aware building control. Qaisar et al. explored large language models for indoor occupancy measurement [10] and demonstrated the energy benefits of occupancy-centric HVAC strategies [11], while OCC-Mamba introduced a deep sequence modeling approach for indoor occupancy prediction [12]. These studies illustrate the increasing use of advanced AI architectures for predictive building control, which in turn requires large and diverse datasets capturing a wide range of operating conditions and control actions.

Robust control-oriented modeling requires data that capture a wide range of building states and control actions. Existing public datasets, while extensive, are based on standard control schedules and lack coverage of exploratory or suboptimal control policies and, as Pinto et al. concluded in [13], there is a need for more comprehensive, large-volume, well-recognized datasets in the building field. This gap motivates our contribution, a dataset that spans six different buildings and four distinct climate zones, operated under two contrasting control policies (rule-based and deliberately stochastic) across one simulated year each at 15-minute resolution, including HVAC cooling and heating setpoints, outdoor weather, indoor temperature, and energy load.

### Method details

The method generates HVAC operation datasets by executing 1 full year building simulations at 15-minute resolution under two supervisory control strategies:

• Default rule-based controller (RBC): the conventional schedule-based control provided by the simulation environments.
• Stochastic exploratory controller: a supervisory policy that randomly alternates between multiple short-term behaviors to generate diverse heating and cooling setpoint trajectories.

For each scenario (building × climate × control policy), the method collects multivariate time series containing indoor temperature, HVAC power, heating/cooling setpoints, and outdoor weather variables. Scenarios are aggregated into dataset splits (training and test) and exported to Parquet format, along with metadata enabling separation of series and policies.

The simulated buildings included in the dataset are summarized in Table 1, Table 2. Five source buildings were simulated using the Sinergym framework. These buildings differ in geometry, floor area, and number of thermal zones. An additional building is simulated using the Boptest framework [14]. To support research on transfer learning, the five buildings simulated using Sinergym are designated as source buildings, from which models are expected to learn transferable patterns, while the building simulated using Boptest is designated as the target building, on which knowledge transfer is evaluated.

**Table 1:** Source buildings geometry.

Building name	Number of zones	Floor area (m²)
5Zone	5	463.6
Warehouse	3	4,598
OfficeMedium	15	4,979.6
ShopWithVanBattery	5	390.2
OfficeGridStorageSmoothing	19	46,320.0

**Table 2:** Target building geometry.

Building name	Number of zones	Floor area (m²)
BestestAir (Case 900)	1	48

Each source building was simulated under three distinct Typical Meteorological Year (TMY3) climates, listed in Table 3, representing hot desert, humid subtropical, and marine west coast conditions. The target building was simulated under a single climate, shown in Table 4, corresponding to a semi-arid continental profile. All simulated buildings have both heating and cooling systems.

**Table 3:** Source climates used for Sinergym simulations.

Location	Climate type	Description
Tucson, AZ	Hot desert	Very hot, dry summers and mild winters; cooling-dominated
New York, NY	Humid subtropical	Hot, humid summers and cold winters with year-round precipitation
Port Angeles, WA	Marine west coast	Cool, cloudy climate with frequent rain; heating-dominated

**Table 4:** Target climate used for Boptest simulations.

Location	Climate type	Description
Denver, CO	Semi-arid continental	Cold winters and hot, dry summers with large daily temperature swings

Two supervisory control policies were applied during simulation. The first is a default rule-based controller provided by the simulation frameworks, representing standard HVAC operation. The second is a deliberately stochastic exploratory policy that generates diverse heating and cooling setpoint trajectories by interleaving multiple short-term behaviours. Both control policies were applied consistently across buildings and climates. More details on the control policies are given in the next section.

Based on the simulated scenarios, multiple dataset splits were created for training and evaluation purposes. An overview of all datasets, including their role, composition, and duration, is provided in Table 5. Training datasets include multi-building source data as well as target-only data generated under individual control policies.

**Table 5:** Training and test datasets overview (15-minute sampling).

Role	Dataset name	Composition	Duration
Train	source-all	5 source buildings × 3 climates × {default, chaotic}	30 years
Train	source-default	5 source buildings × 3 climates × {default}	15 years
Train	target-default	1 target building × 1 climate × {default}	10 months
Train	target-chaotic	1 target building × 1 climate × {chaotic}	10 months
Test	target-heat-test	1 target building × 1 climate × {mixed policy}	1 month
Test	target-cool-test	1 target building × 1 climate × {mixed policy}	1 month

In Table 5, “years” refers to full calendar years of continuous simulation at 15-minute sampling (Jan 1 00:00 to Dec 31 23:45) for each (building × climate × control policy) scenario. For the target domain, the released training sets (target-default, target-chaotic) contain 10 months of data and exclude the two test months used to evaluate seasonal performance. The target test sets are fixed calendar windows to ensure reproducibility: January is used for heating evaluation (target-heat-test) and July for cooling evaluation (target-cool-test). We do not perform an explicit warm-up period, the exported time series starts at the simulator’s default initial conditions and includes the complete year. For reproducibility, random seeds are fixed per scenario (building × climate × control policy × year), enabling deterministic regeneration of the controller trajectories and resulting datasets.

In both target test sets, the controller applied during simulation alternates between the default rule-based controller (RBC) and the stochastic policy within the same continuous month-long trajectory. The switching schedule is predefined in configuration and fixed across runs for reproducibility. The rationale for this mixed-policy design is to evaluate model robustness under supervisory-policy changes without resetting the system state, thereby testing adaptation across distinct action distributions within a single thermodynamically continuous trajectory.

Each dataset is a single multivariate time series or a collection of concatenated time series sampled at 15-minute resolution. The following variables are present in all datasets:

All variables are stored using physical units as indicated in their column names. No normalization, aggregation, or rescaling is applied to the released data. HVAC Power Consumption measured in Watts (W) represents instantaneous average electrical HVAC power over the 15-min timestep, reported at environment level (whole building). Includes all HVAC electricity exposed by the simulator interface. For multi-zone EnergyPlus buildings, Indoor Air Temperature is the arithmetic mean of zone air temperatures in °C; for single-zone models it is the zone temperature.

The exported variable set is intentionally minimal and restricted to signals that are (i) consistently available across all simulated buildings and (ii) comparable across both simulation stacks used in this work (EnergyPlus/Sinergym and Modelica/BOPTEST) [15]. In practice, many additional control relevant variables (e.g., component-level power breakdown (fans/coils/pumps), airflow rates, valve/damper commands, zone humidity/latent loads, or internal gains/occupancy indicators) are model dependent and not uniformly exposed across environments. Including them in the default release would therefore reduce cross-building and cross-simulator compatibility and complicate reproducible benchmarking. The chosen schema preserves a consistent interface focused on the core supervisory control loop, while still supporting common tasks such as system identification, evaluation under diverse setpoint trajectories, and transfer-learning evaluation and reproducible comparisons.

To make the release directly usable for transfer-learning studies, we recommend the following tasks that can be instantiated from the common schema (Table 6) under both supervisory policies (RBC and stochastic):

• One-step / multi-step prediction: forecast Room Air Temperature and/or HVAC Power from past observations, weather, and setpoints (with configurable horizons).
• Power prediction under setpoint changes: predict HVAC Power Consumption given weather, temperature history, and setpoint trajectories (useful for demand-aware control).

**Table 6:** Common variables description.

**Variable name**	**Type**	**Description**
Room Air Temperature (°C)	float64	Indoor air temperature
Outdoor Air Temperature (°C)	float64	Outdoor dry-bulb temperature
Outdoor Humidity (%)	float64	Outdoor relative humidity
Direct Solar Radiation (W/m²)	float64	Direct normal solar radiation
Wind Speed (m/s)	float64	Outdoor wind speed
Cooling Setpoint (°C)	float64	Cooling temperature setpoint
Heating Setpoint (°C)	float64	Heating temperature setpoint
HVAC Power Consumption (W)	float64	Electrical power consumption of the HVAC system

All datasets were generated using open-source building simulation frameworks. For source buildings, we used Sinergym v3.9.5, which exposes a Gym-compatible Python interface. For the target building, we used BOPTEST v0.7.1 patched with the fix described in issue 769, together with BOPTEST-Gym v0.7.0, which provides a unified Gym interface. These tools enable simple data collection directly from Python.

Both simulators were controlled from Python through their Gym-compatible environments (Sinergym itself and BOPTEST-Gym for BOPTEST). The Sinergym environment IDs used in this work correspond to the five source buildings listed in Table 1 (5Zone, Warehouse, OfficeMedium, ShopWithVanBattery, OfficeGridStorageSmoothing). For the target simulator, we used the BOPTEST “bestest_air” testcase (BestestAir / Case 900, Table 2) exposed via BOPTEST-Gym. In both cases, actions/observations are exchanged through the standard Gym step() loop. We use the default configurations shipped with each environment, while the released code includes the exact observation/action mapping used to build the standardized dataset columns in Table 6 for both simulators.

In this workflow, EnergyPlus (via Sinergym) and Modelica (via BOPTEST) are not executed in a coupled manner. Each building scenario is simulated independently within its respective engine, and datasets are generated separately. The common Gym-compatible interface provides a standardized interaction layer (observations, actions, and logging), but no runtime exchange of variables occurs between the two simulation engines.

To make Sinergym (EnergyPlus) and BOPTEST (Modelica) outputs comparable, we export a common set of variables with fixed names and units (Table 6): temperatures in °C, outdoor RH in %, direct normal solar radiation in W/m², wind speed in m/s, and HVAC electrical power in W. At each 15 min step, the supervisory controller outputs two scalar actions (Heating Setpoint (°C) and Cooling Setpoint (°C)) which are mapped to the simulators’ zone/thermostat setpoint inputs (Sinergym: thermostat schedule/setpoint actuators; BOPTEST: zone temperature setpoint inputs). When a model has multiple zones, the exported Room Air Temperature (°C) corresponds to the environment’s standard “zone temperature” observation (single-zone for BestestAir; for multi-zone EnergyPlus buildings, the zone temperature is computed as the average of each zone temperature. HVAC Power Consumption (W) is the total HVAC electrical power reported by each environment (aggregated at environment level),

Both Sinergym and BOPTEST are accessed through a standard Gym-like loop: reset() returns the initial observation at the start of the episode; then at each 15-min step the controller computes two supervisory actions (Heating Setpoint (°C) and Cooling Setpoint (°C)) which are written to the environment action interface (Sinergym: thermostat setpoint actuators; BOPTEST: zone temperature setpoint inputs). The environment then advances one simulation step and returns (observation, reward, done, info). We log the observation channels listed in Table 6 (Room Air Temperature, weather variables, setpoints, HVAC Power) at every step; reward/done/info are used only for stepping/termination and are not part of the released dataset schema.

All simulations are executed with a 15-minute control step, matching the released dataset sampling resolution. In both Sinergym (EnergyPlus) and BOPTEST (Modelica), the supervisory controller updates the heating/cooling setpoints once every 15 minutes, and the simulator returns observations and HVAC power at the same 15-minute timestamps. This produces aligned, regular time indices across engines (no resampling or post-hoc aggregation is applied).

To estimate the computational effort required to generate one full year of simulated data per scenario, all simulations were executed using the following setup:

• Compute node: Google Cloud e2-standard-4 (4 vCPUs, 16 GB RAM; Intel Broadwell, x86_64)
• Host operating system: Ubuntu 25.04
• Container base image: Ubuntu 22.04 (Docker)

The tables below report the execution time required to simulate one full year of building operation at a 15-minute sampling resolution. Modelica based BOPTEST simulations require substantially more computation time, as they resolve more detailed physical models of both the building envelope and HVAC systems. In contrast, EnergyPlus based Sinergym simulations rely on predefined component models and execute significantly faster. This difference in simulation fidelity motivated the use of BOPTEST as the target domain (Table 7, 8).

**Table 7:** Execution times for Boptest simulations.

Test name	Control policy	Duration
bestest_air	Stochastic	03 h 52 m 54.7 s
bestest_air	Default	02 h 36 m 37.0 s

**Table 8:** Execution times for Sinergym simulations.

**Test name**	**Control policy**	**Duration**
5zone	Stochastic	00 m 40 s
5zone	Default	00 m 29 s
office	Stochastic	02 m 07 s
office	Default	01 m 43 s
officegrid	Stochastic	02 m 03 s
officegrid	Default	01 m 33 s
shop	Stochastic	00 m 55 s
shop	Default	00 m 36 s
warehouse	Stochastic	01 m 11 s
warehouse	Default	00 m 43 s

We designate BOPTEST as the target domain due to fidelity and computational cost differences (more detailed Modelica-based physics), rather than any coupling between simulation engines.

The default rule-based control (RBC) schedules for each building simulated with Sinergym are summarized in Table 9, while Table 10 shows those simulated with Boptest. These schedules define occupied and unoccupied periods and the corresponding heating and cooling temperature setpoint

**Table 9:** Sinergym (EnergyPlus) source buildings and default RBC schedules.

Building	Geometry	Occupied hours / setpoints (°C)	Unoccupied hours / setpoints (°C)
5Zone		06:45–17:30 / [21.1, 23.9]	17:45–06:30 / [12.8, 40.0]
Warehouse		04:45–16:30 / [21.1, 23.9]	16:45–04:30 / [15.6, 29.4]
OfficeMedium		05:45–20:30 / [21, 24]	20:45–05:30 / [15.6, 26.7]
ShopWithVanBattery		08:45–17:30 / [21, 24]	17:45–08:30 / [9, 33]
OfficeGridStorage		04:45–20:30 / [21, 24]	20:45–04:30 / [15.6, 26.7]

**Table 10:** BOPTEST (Modelica) target building and default RBC schedule.

Building	Geometry	Occupied hours / setpoints (°C)	Unoccupied hours / setpoints (°C)
BestestAir		08:00–17:45 / [21, 24]	18:00–07:45 / [10, 30]

The stochastic policy is a supervisory setpoint generator that produces diverse setpoint trajectories while enforcing operational bounds. At runtime, it alternates between a set of short-horizon behaviors:

• Ornstein–Uhlenbeck drift: random walk around a target with mean reversion.
• Ramp: gradual increase/decrease with random direction changes.
• Oscillation: sinusoidal oscillations with additive noise.
• Jump: abrupt setpoint change to a new random value.
• Hold: constant setpoints with small perturbations.

Switching rule: every *K* steps, where *K* is randomly sampled in a predefined interval (e.g., 10–100 steps), the controller selects a behavior uniformly (or via conFig.d probabilities). In all experiments, heating and cooling setpoints are clipped to fixed operational bounds consistent with the underlying building models. In addition, the implementation supports an optional minimum deadband (i.e., minimum separation between heating and cooling setpoints) to avoid overlap (which we did not use no our experiments). In the released datasets, the default configuration preserves the native simulator-level thermostat logic (EnergyPlus/BOPTEST), which internally enforces physically consistent operation and prevents simultaneous heating and cooling conflicts. Because the control resolution is 15 minutes, no additional anti-chatter or dwell-time logic was required in practice, and no pathological high-frequency switching was observed in the resulting trajectories.

High-level implementation:

1. Initialize setpoints within bounds; set random seed for reproducibility.

2. For each time step:

• If remaining duration in current behavior is 0: sample a new behavior and new duration.
• Update heating and cooling setpoints according to the behavior dynamics.
• Clip setpoints to bounds; optionally enforce minimum separation between heating and cooling.
• Output setpoints to the simulator; log resulting state and power.

The stochastic policy is intentionally allowed to generate non routine setpoint combinations to broaden action–state coverage. In practice, both simulation stacks enforce feasibility at the actuator level: setpoint commands are clipped to conFig.d bounds before being applied, and the underlying simulators (EnergyPlus via Sinergym; Modelica via BOPTEST) enforce additional model-specific operational limits (e.g., controller/actuator constraints). As a result, extreme requests do not lead to unbounded behavior; they either saturate at the imposed limits or yield physically consistent responses within the model. We monitor simulation completion and log simulator messages; across the published runs we did not observe systematic simulation failures attributable to the exploratory policy.

The released code contains exact hyperparameters (noise scales, ramp rates, oscillation amplitudes/periods, bounds, and seed handling), enabling reproduction of the published datasets. Table 11, Table 12 summarize the parameter ranges and dataset specific bounds used in the experiments.

**Table 11:** Parameterization of chaotic HVAC supervisory setpoint primitives. Distributions are sampled at primitive initialization unless otherwise stated. All values are applied in the native units of the simulation environment (°C in Sinergym source datasets; K in BOPTEST target datasets).

Primitive	Parameter	Exact Value / Distribution	When Applied
OUBehavior (Mean-Reverting Drift)	Cooling mean target	μ_c ∼ Uniform(cool_min, cool_max)	Once per instance
	Heating mean target	μ_h ∼ Uniform(heat_min, heat_max)	Once per instance
	Mean reversion rate	θ ∼ Uniform(0.05, 0.5)	Once per instance
	Noise scale	σ ∼ Uniform(0.5, 2.0)	Once per instance
	Time increment	dt = 1.0	Each step
	Process noise	ε ∼ Normal(0, 1), scaled by σ	Each step
RampBehavior (Gradual Drift + Turns)	Ramp increment	δ ∼ Uniform(0.2, 1.0) × direction	Each step
	Initial direction	direction = +1	At initialization
	Turn probability	p_turn ∼ Uniform(0.05, 0.30)	At initialization
OscillationBehavior (Sinusoidal)	Initial phase	φ ∼ Uniform(0, 2π)	Once per instance
	Frequency	f ∼ Uniform(0.01, 0.1)	Once per instance
	Amplitude	A ∼ Uniform(1.0, 5.0)	Once per instance
	Phase update	φ ← φ + f + Normal(0, 0.1·f)	Each step
	Relative phase offset (heat vs cool)	Δφ ∼ Uniform(0, π/2)	Once per instance
	Additive jitter	Normal(0, 0.3)	Each step
StepJumpBehavior (Abrupt Redraw) HoldWithNoiseBehavior	Cooling setpoint	Uniform(cool_min, cool_max)	Each step in behavior
	Heating setpoint	Uniform(heat_min, heat_max)	Each step in behavior
	Additive noise	Normal(0, 0.3)	Each step
	Ordering enforcement	Cooling ≥ Heating via swap(max, min)	Every step

**Table 12:** Dataset-specific supervisory setpoint bounds and random seeds used for chaotic policy generation. Cooling and heating bounds define hard constraints applied to all primitives. Seeds ensure full reproducibility of trajectory generation.

Script / Dataset	Units	Cooling Bounds (min, max)	Heating Bounds (min, max)	Seed
run_office.py	°C	(22.5, 30.0)	(15.0, 22.5)	42
run_5zone.py	°C	(23.25, 30.0)	(12.0, 23.25)	42
run_shop.py	°C	(22.5, 35.0)	(10.0, 22.5)	42
run_warehouse.py	°C	(22.5, 35.0)	(15.0, 22.5)	42
run_bestest_air.py (BOPTEST)	K	(295.65, 308.15)	(283.15, 295.65)	42

### Method validation

We validate the method along two dimensions: (i) functional reproducibility and (ii) action-space enrichment achieved by the stochastic controller.

Regarding functional reproducibility, the full simulation and dataset assembly pipeline is released with a DOI and includes pinned dependency versions, configuration files, and scripts to regenerate every published dataset split. Each dataset repository provides loading examples and unit-consistent columns.

**Fig. 1:**
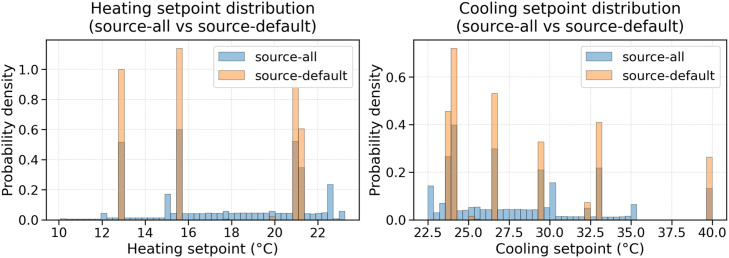
Value distribution for control variables on source-all dataset compared to source-default.

A core goal of the method is to enrich action-state coverage beyond routine RBC schedules. Under RBC-only operation, heating and cooling setpoints concentrate at a small number of scheduled values, providing a narrow distribution. By design, the stochastic controller produces trajectories with ramps, oscillations, drifts, and jumps, resulting in broader distributions for both heating and cooling setpoints while remaining within operational bounds. Fig. 1 below shows a comparison of the value distribution for cooling and heating setpoints between source-all dataset and source-default dataset.

**Fig. 2:**
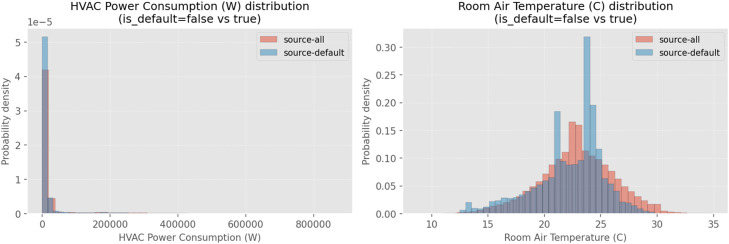
Value distribution for state variables on source-alll dataset compared to source-default.

To assess whether this action diversity translates into richer state and response coverage, we additionally compare distributions of indoor air temperature and HVAC power consumption. Fig. 2 shows that the exploratory controller expands the support of indoor temperature and HVAC power distributions relative to RBC operation. In particular, indor air temperature exhibits a wider dynamic range and increased occupancy of intermediate operating regions, indicating excitation of part-load and transient behaviors that are underrepresented in routine schedules.

This validation supports the method’s intended use for robustness and transfer-learning research, where diversity of supervisory control inputs is critical (Table 13).

**Table 13:** Quantitative action–state coverage metrics (mean ± std over 15 one-year source scenarios).

Metrics	Default (RBC)	Stochastic	JSD (RBC vs Stochastic)
H(Heating setpoint) [bits]	1.03 ± 0.18	3.92 ± 0.38	0.78 ± 0.05
H(Cooling setpoint) [bits]	1.12 ± 0.24	3.96 ± 0.39	0.81 ± 0.07
H(Heating, Cooling joint) [bits]	1.13 ± 0.22	7.56 ± 0.64	0.98 ± 0.01
C(Heating, Cooling) [% bins visited]	0.8% ± 0.5%	88.8% ± 23.1%	—
H(Room Temp, Power_norm) [bits]	6.26 ± 0.35	7.52 ± 0.47	0.28 ± 0.05
C(Room Temp, Power_norm) [% bins visited]	24.8% ± 5.6%	51.7% ± 9.8%	—
Joint coverage (Action + State) [% of union bins]	2.5% ± 0.8%	97.9% ± 0.8%	—

In this work, action–state coverage refers to how broadly the dataset explores the joint space of supervisory control actions (heating and cooling setpoints) and resulting system states (indoor temperature and HVAC power consumption).

To quantify coverage beyond visual histograms, we compute reproducible bin-based metrics between default and chaotic time-series. Metrics used are:

1. Shannon entropy (H) to measure distributional diversity: H = − Σ pᵦ log₂ pᵦ, where pᵦ is the empirical probability of bin b.
2. Support coverage (C) defined as the fraction of bins visited: C = (number of bins with at least one sample) / (total bins).
3. Jensen–Shannon divergence (JSD) between RBC and chaotic distributions to quantify distributional shift.

The three measures capture complementary aspects of how broadly the dataset explores possible operating conditions. Shannon entropy (H) measures how spread out a variable’s values are across the defined bins: higher entropy indicates that the system visits many different values rather than concentrating on only a few typical ones. Support coverage (C) measures how much of the possible range of values is actually visited, expressed as the fraction of bins that contain at least one observation. Finally, Jensen–Shannon divergence (JSD) quantifies how different two distributions are, in this case, the distributions produced by the default rule-based controller and the stochastic controller. Larger JSD values indicate that the exploratory controller produces behavior that is substantially different from the routine scheduled control.

For comparability across buildings and climates:

• Heating, cooling setpoints and indoor temperature are discretized using 0.5 °C bins.
• HVAC power is normalized per scenario by the 95th percentile (P_norm = P / P95, clipped to [0, 16]) and discretized using 0.05 bins.
• Metrics are computed per one-year (building × climate) scenario and reported as mean ± standard deviation across all 15 source scenarios.

The results quantitatively confirm the action–state enrichment introduced by the stochastic controller. Entropy of heating and cooling setpoints increases by more than 3x relative to RBC, and joint action entropy increases from 1.13 bits to 7.56 bits, indicating substantially broader exploration of the supervisory action space. Action-bin support coverage expands from less than 1% under RBC to nearly 89% under chaotic control.

This way action diversity propagates to the physical system response. Joint temperature–power entropy increases, and state support coverage approximately doubles (24.8% to 51.7%), indicating excitation of operating regions that are rarely visited under schedule-based control. The near complete joint action–state coverage (97.9% of union bins) under stochastic operation further demonstrates that the method systematically expands the explored operating manifold rather than merely perturbing scheduled setpoints.

Taken together, these results provide quantitative evidence that the proposed controller not only increases action diversity but also broadens the range of system states that are visited, reinforcing its suitability as a mechanism for generating rich trajectories and transfer-learning evaluations.

All simulations are executed with fixed random seeds (see Table 12) and pinned versions of Sinergym, EnergyPlus, BOPTEST, and their dependencies. The stochastic controller is fully seed controlled and does not rely on any non-deterministic runtime sources. Under identical software versions and configuration files, re-running the pipeline yields identical time series. Because the workflow relies on deterministic building simulation engines (EnergyPlus and Modelica-based BOPTEST), no stochastic numerical variance is expected across runs. Minor floating-point differences could in principle arise if different solver versions or compiler builds are used; however, with the provided containerized environment and pinned versions, outputs are reproducible without observed numerical drift.

### Limitations

The proposed workflow generates HVAC operation time series using established physics-based building simulation engines. While this enables controlled experimentation, repeatability, and systematic coverage across buildings, climates, and supervisory control trajectories, the resulting signals reflect the assumptions and level of detail of the underlying simulators and their conFig.d models. Consequently, some operational phenomena that arise in deployed buildings may not be fully represented (e.g., unmodeled equipment faults, commissioning drift, or site-specific control idiosyncrasies). The supervisory controller operates at a 15-minute resolution and relies on simulator-level HVAC actuation logic for physical feasibility; it is not intended to represent deployable real-time equipment-level control safeguards such as compressor cycling limits or minimum on/off dwell constraints.

In addition, the amount of scenarios is bounded by the selected set of building models, climates, and supervisory strategies included in this release. The method is designed to be extensible, and can be expanded by adding further building typologies, HVAC configurations, climates, and alternative supervisory controllers within the same pipeline.

While we keep a minimal common schema for comparability, the released pipeline is extensible: additional outputs can be enabled when they are available in a given building model/simulator by extending the variable-mapping configuration. Examples of planned/possible extensions include (i) HVAC sub-metering or component power breakdown, (ii) supply/zone airflow and actuator states (e.g., valve/damper signals), (iii) zone humidity variables, and (iv) occupancy schedules or internal gains when explicitly defined. These extensions are intentionally not part of this work because their definition and availability vary across building models and simulation engines.

## Ethics statements

Our study does not involve studies with animals or humans. Therefore, we confirm that our research strictly adheres to the guidelines for authors provided by MethodsX in terms of ethical considerations.

## CRediT author statement

**Ferran Aran Domingo:** Conceptualization, Methodology, Software, Data curation, Investigation, Writing – Original Draft. **Pablo Fraile Alonso:** Conceptualization, Methodology, Supervision. **Josep Rius Torrentó:** Supervision, Writing – Review & Editing. **Oriol Agost Batalla:** Software, Writing – Review & Editing. **Ignasi Barri Vilardell:** Resources. **Jordi Vilaplana Mayoral:** Supervision, Project administration. **Jordi Mateo Fornés:** Supervision.

Supplementary material *and/or* additional information [OPTIONAL]

Code repository (GitHub): https://github.com/dcg-udl-cat/ttm4hvac, which includes:

• Scripts to run Sinergym and BOPTEST simulations.
• Stochastic controller implementation.
• Configurations specifying buildings, climates, seeds, horizons.
• Devcontainers to reproduce the environments where the simulations were executed.

Code DOI: https://doi.org/10.5281/zenodo.18378593

Dataset DOI: https://doi.org/10.57967/hf/7507

Datasets (HuggingFace):

• https://huggingface.co/datasets/gft/ttm4hvac-source-all-train
• https://huggingface.co/datasets/gft/ttm4hvac-source-default-train
• https://huggingface.co/datasets/gft/ttm4hvac-target-default-train
• https://huggingface.co/datasets/gft/ttm4hvac-target-stochastic-train
• https://huggingface.co/datasets/gft/ttm4hvac-target-heat-test
• https://huggingface.co/datasets/gft/ttm4hvac-target-cool-test

## Declaration of interest

The authors declare that they have no known competing financial interests or personal relationships that could have appeared to influence the work reported in this paper.

## Data Availability

The dataset is made public and free to access on HugginFace.
